# Supplementary material for: Canopy position has a profound effect on soybean seed composition
Source: PeerJ. 2016 Sep 13;4:e2452. doi: 10.7717/peerj.2452 (PMC5028787; doi:10.7717/peerj.2452)
Supplement: File S1 — Values are PPM (Elements), mg (SampleWeight), and Percentage (Protein/Oil). [file peerj-04-2452-s001.pdf]

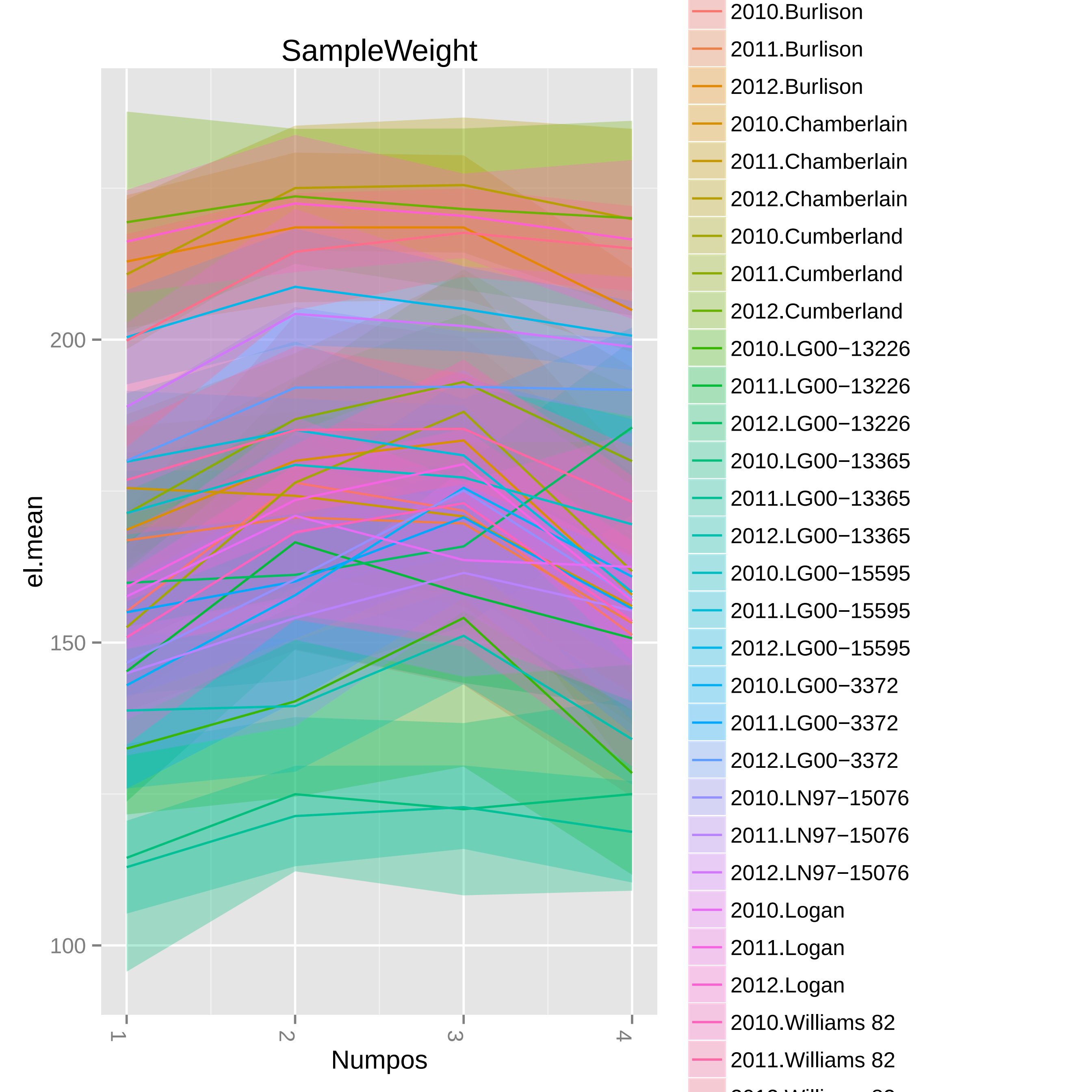
SampleWeight
el.mean
200
150
100
Numpos
1
2
3
4
2010.Burlison
2011.Burlison
2012.Burlison
2010.Chamberlain
2011.Chamberlain
2012.Chamberlain
2010.Cumberland
2011.Cumberland
2012.Cumberland
2010.LG00−13226
2011.LG00−13226
2012.LG00−13226
2010.LG00−13365
2011.LG00−13365
2012.LG00−13365
2010.LG00−15595
2011.LG00−15595
2012.LG00−15595
2010.LG00−3372
2011.LG00−3372
2012.LG00−3372
2010.LN97−15076
2011.LN97−15076
2012.LN97−15076
2010.Logan
2011.Logan
2012.Logan
2010.Williams 82
2011.Williams 82

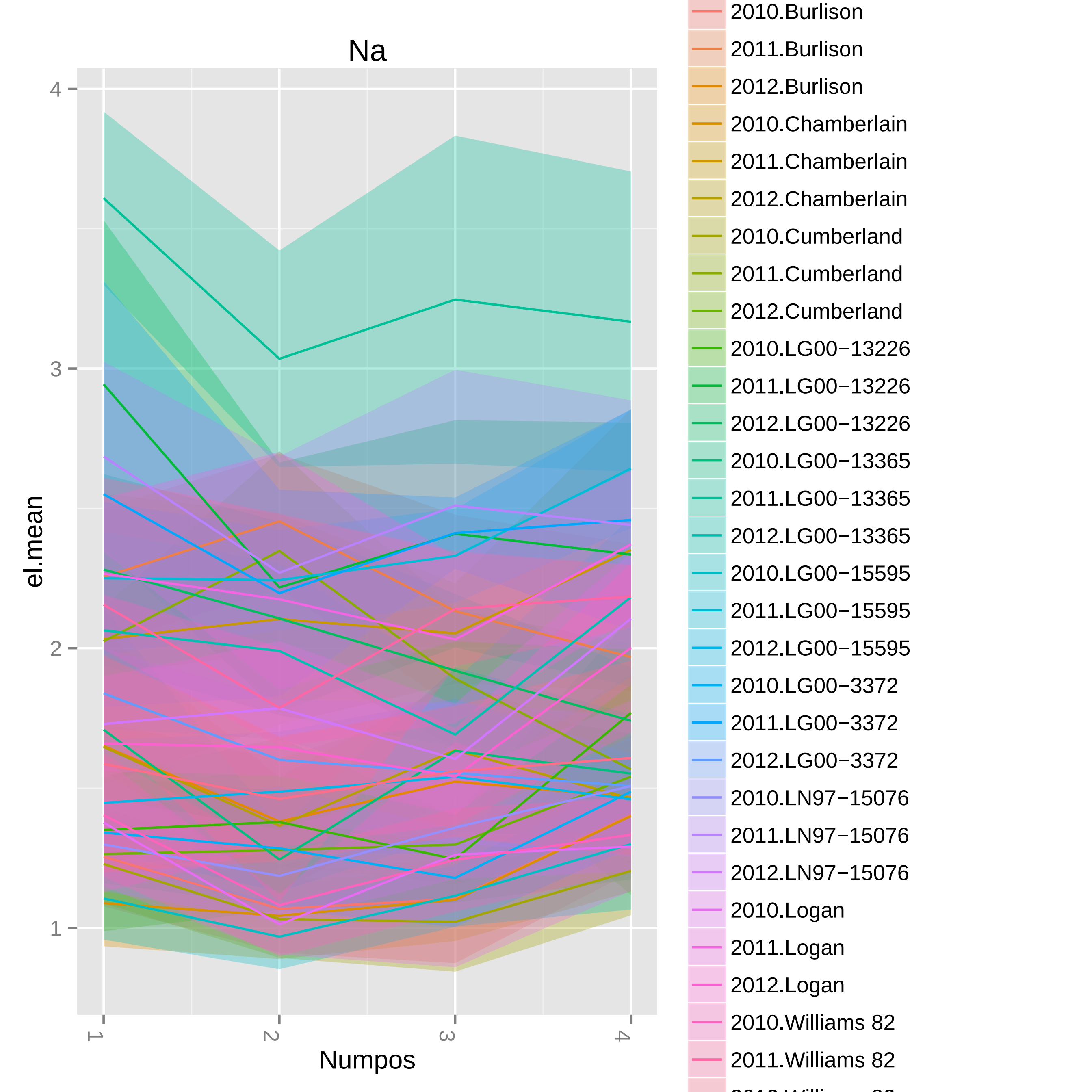
Na
el.mean
Numpos
1
2
3
4
2010.Burlison
2011.Burlison
2012.Burlison
2010.Chamberlain
2011.Chamberlain
2012.Chamberlain
2010.Cumberland
2011.Cumberland
2012.Cumberland
2010.LG00−13226
2011.LG00−13226
2012.LG00−13226
2010.LG00−13365
2011.LG00−13365
2012.LG00−13365
2010.LG00−15595
2011.LG00−15595
2012.LG00−15595
2010.LG00−3372
2011.LG00−3372
2012.LG00−3372
2010.LN97−15076
2011.LN97−15076
2012.LN97−15076
2010.Logan
2011.Logan
2012.Logan
2010.Williams 82
2011.Williams 82

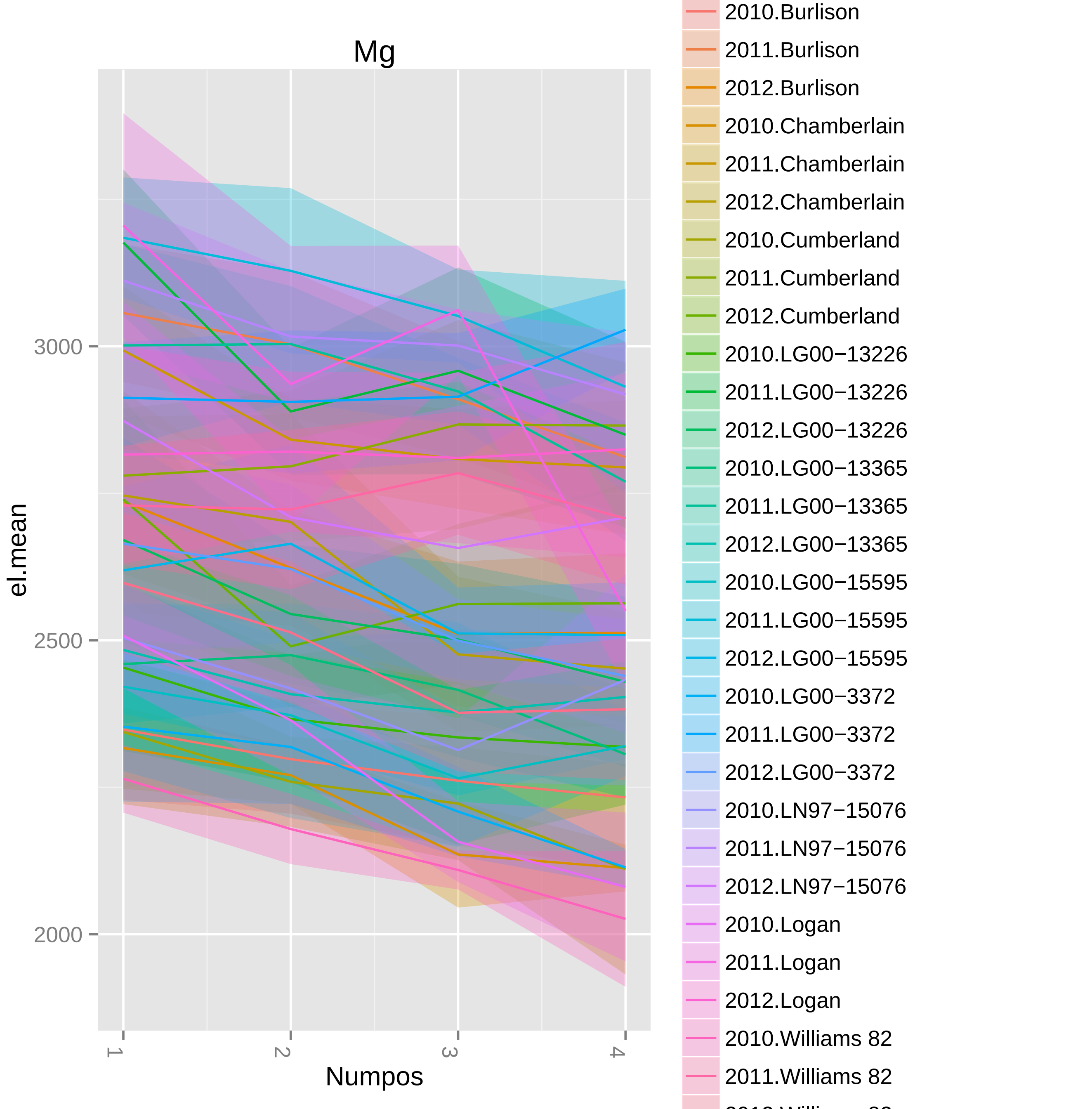

Mg
el.mean
3000
2500
2000
Numpos
1
2
3
4
2010.Burlison
2011.Burlison
2012.Burlison
2010.Chamberlain
2011.Chamberlain
2012.Chamberlain
2010.Cumberland
2011.Cumberland
2012.Cumberland
2010.LG00−13226
2011.LG00−13226
2012.LG00−13226
2010.LG00−13365
2011.LG00−13365
2012.LG00−13365
2010.LG00−15595
2011.LG00−15595
2012.LG00−15595
2010.LG00−3372
2011.LG00−3372
2012.LG00−3372
2010.LN97−15076
2011.LN97−15076
2012.LN97−15076
2010.Logan
2011.Logan
2012.Logan
2010.Williams 82
2011.Williams 82

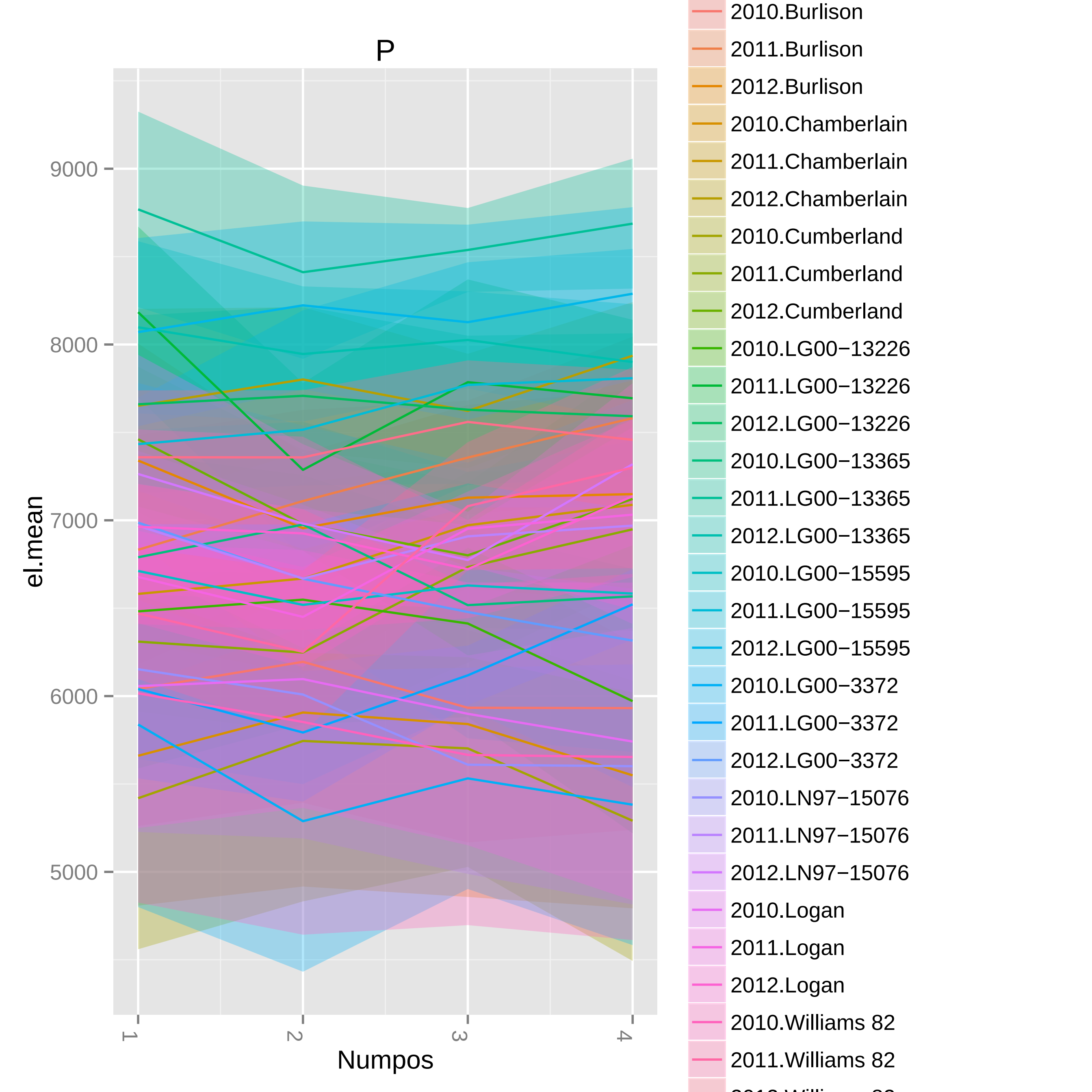

P
el.mean
9000
8000
7000
6000
5000
1
2
3
4
Numpos
2010.Burlison
2011.Burlison
2012.Burlison
2010.Chamberlain
2011.Chamberlain
2012.Chamberlain
2010.Cumberland
2011.Cumberland
2012.Cumberland
2010.LG00−13226
2011.LG00−13226
2012.LG00−13226
2010.LG00−13365
2011.LG00−13365
2012.LG00−13365
2010.LG00−15595
2011.LG00−15595
2012.LG00−15595
2010.LG00−3372
2011.LG00−3372
2012.LG00−3372
2010.LN97−15076
2011.LN97−15076
2012.LN97−15076
2010.Logan
2011.Logan
2012.Logan
2010.Williams 82
2011.Williams 82

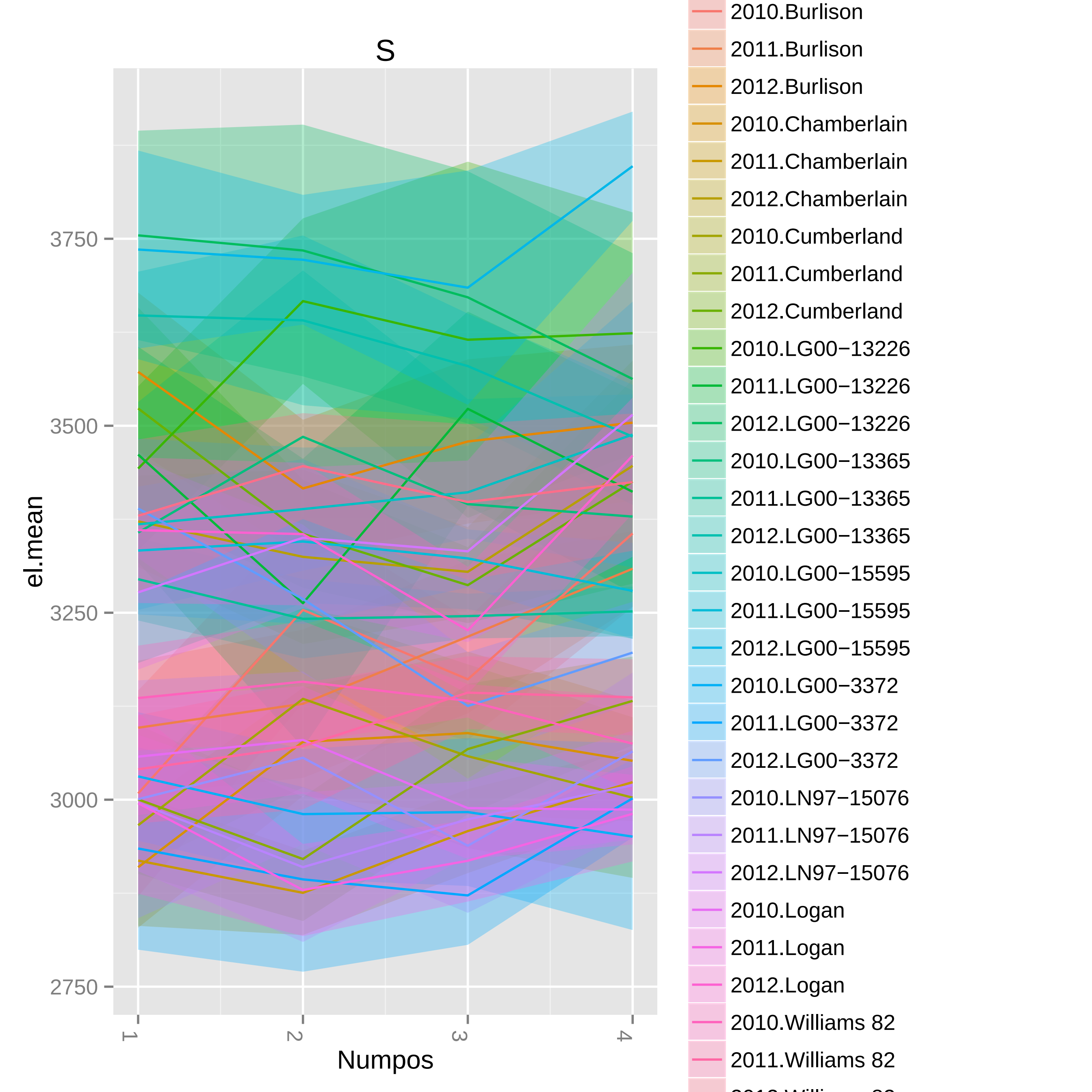
S
el.mean
3750
3500
3250
3000
2750
1
2
3
4
Numpos
2010.Burlison
2011.Burlison
2012.Burlison
2010.Chamberlain
2011.Chamberlain
2012.Chamberlain
2010.Cumberland
2011.Cumberland
2012.Cumberland
2010.LG00−13226
2011.LG00−13226
2012.LG00−13226
2010.LG00−13365
2011.LG00−13365
2012.LG00−13365
2010.LG00−15595
2011.LG00−15595
2012.LG00−15595
2010.LG00−3372
2011.LG00−3372
2012.LG00−3372
2010.LN97−15076
2011.LN97−15076
2012.LN97−15076
2010.Logan
2011.Logan
2012.Logan
2010.Williams 82
2011.Williams 82

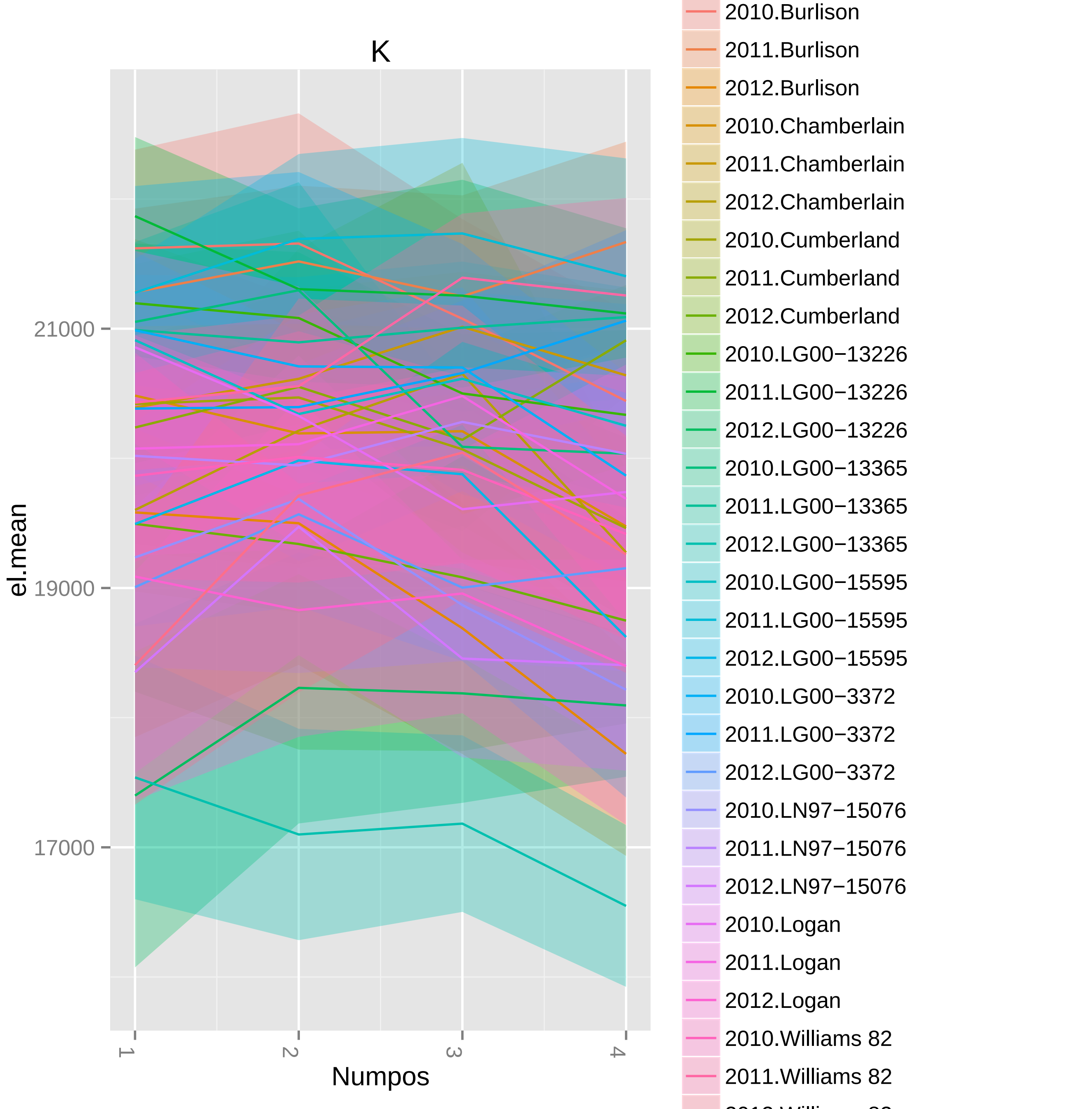

K
el.mean
21000
19000
17000
1
2
3
4
Numpos
2010.Burlison
2011.Burlison
2012.Burlison
2010.Chamberlain
2011.Chamberlain
2012.Chamberlain
2010.Cumberland
2011.Cumberland
2012.Cumberland
2010.LG00−13226
2011.LG00−13226
2012.LG00−13226
2010.LG00−13365
2011.LG00−13365
2012.LG00−13365
2010.LG00−15595
2011.LG00−15595
2012.LG00−15595
2010.LG00−3372
2011.LG00−3372
2012.LG00−3372
2010.LN97−15076
2011.LN97−15076
2012.LN97−15076
2010.Logan
2011.Logan
2012.Logan
2010.Williams 82
2011.Williams 82

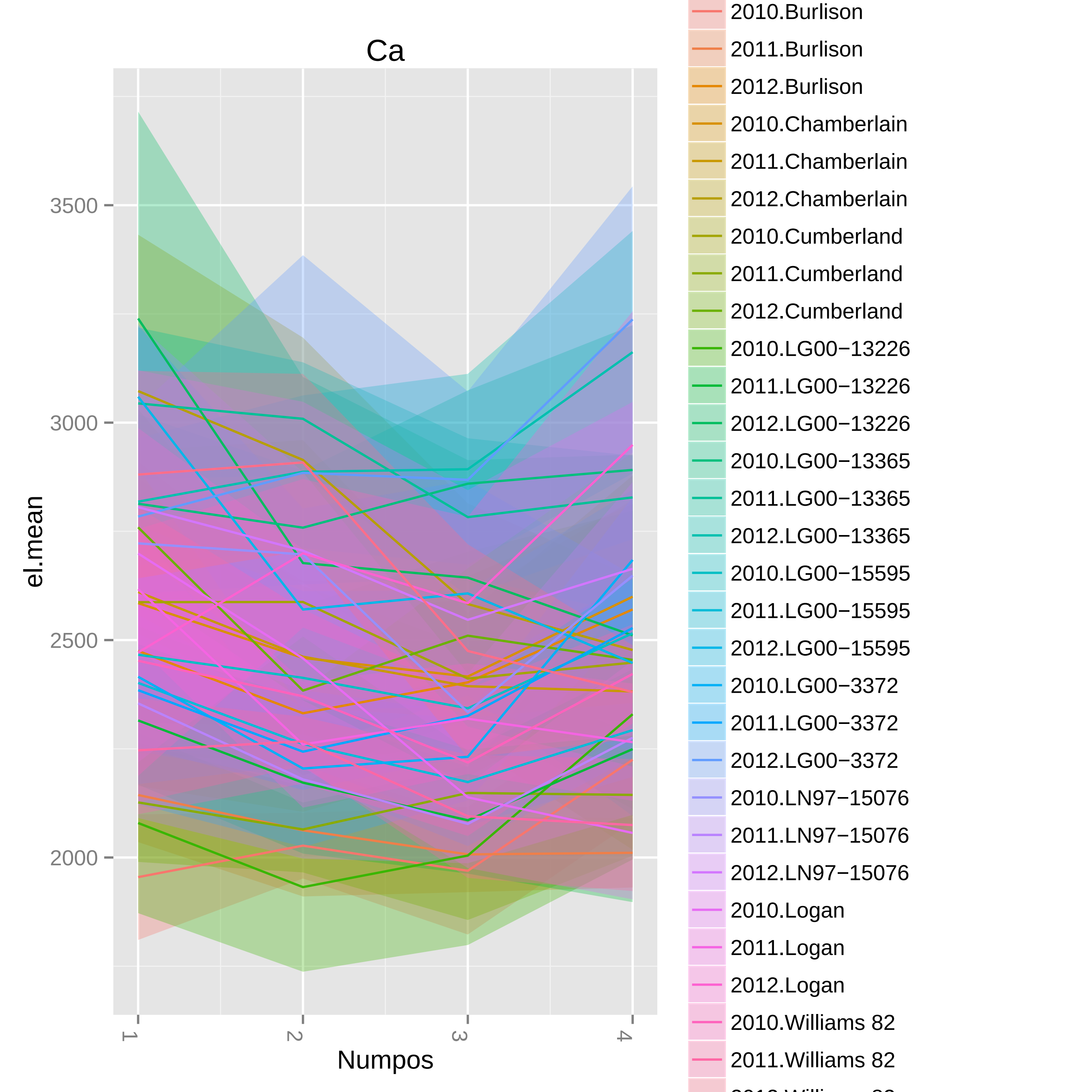

Ca
el.mean
3500
3000
2500
2000
1
2
3
4
Numpos
2010.Burlison
2011.Burlison
2012.Burlison
2010.Chamberlain
2011.Chamberlain
2012.Chamberlain
2010.Cumberland
2011.Cumberland
2012.Cumberland
2010.LG00−13226
2011.LG00−13226
2012.LG00−13226
2010.LG00−13365
2011.LG00−13365
2012.LG00−13365
2010.LG00−15595
2011.LG00−15595
2012.LG00−15595
2010.LG00−3372
2011.LG00−3372
2012.LG00−3372
2010.LN97−15076
2011.LN97−15076
2012.LN97−15076
2010.Logan
2011.Logan
2012.Logan
2010.Williams 82
2011.Williams 82

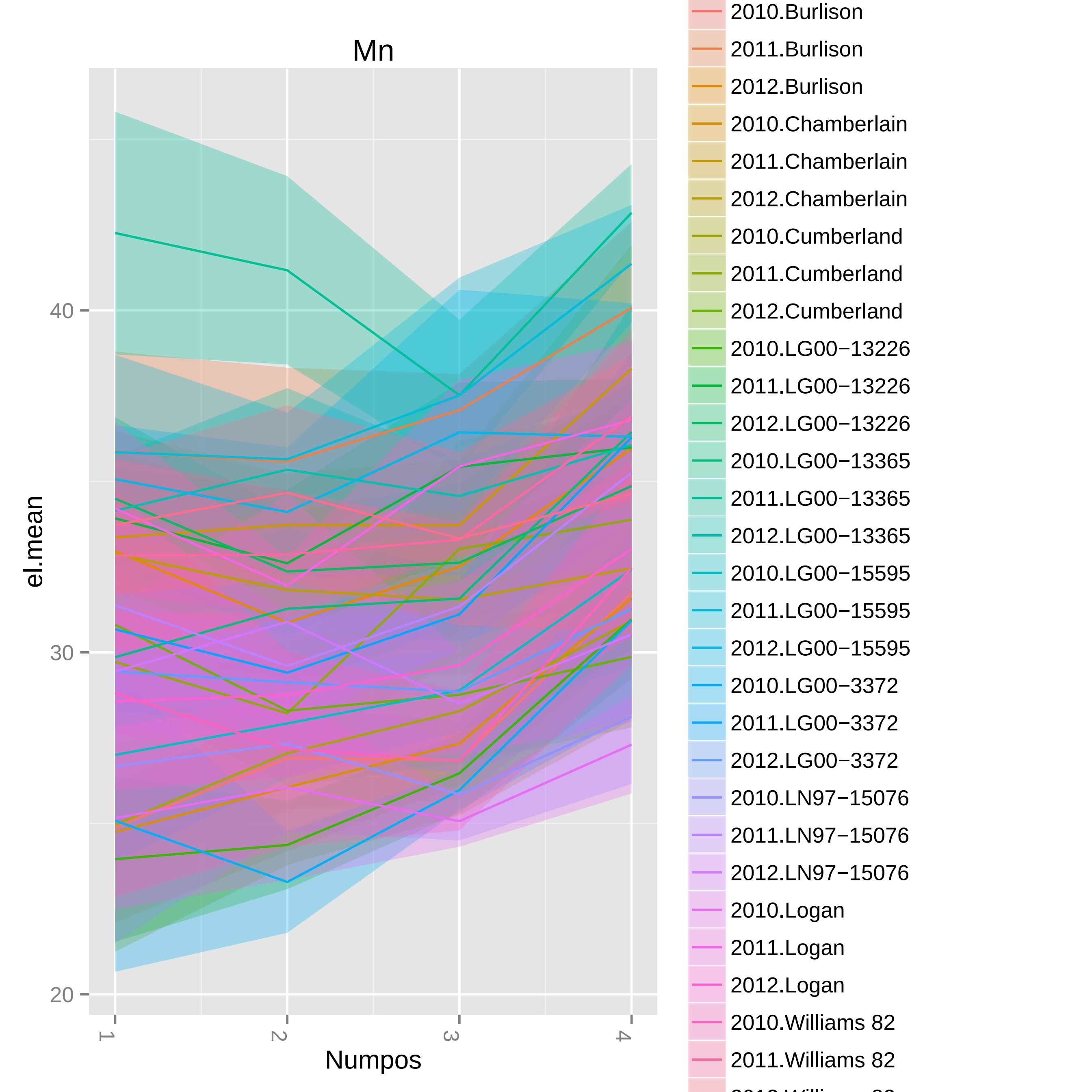
Mn
el.mean
40
30
20
1
2
3
4
Numpos
2010.Burlison
2011.Burlison
2012.Burlison
2010.Chamberlain
2011.Chamberlain
2012.Chamberlain
2010.Cumberland
2011.Cumberland
2012.Cumberland
2010.LG00−13226
2011.LG00−13226
2012.LG00−13226
2010.LG00−13365
2011.LG00−13365
2012.LG00−13365
2010.LG00−15595
2011.LG00−15595
2012.LG00−15595
2010.LG00−3372
2011.LG00−3372
2012.LG00−3372
2010.LN97−15076
2011.LN97−15076
2012.LN97−15076
2010.Logan
2011.Logan
2012.Logan
2010.Williams 82
2011.Williams 82

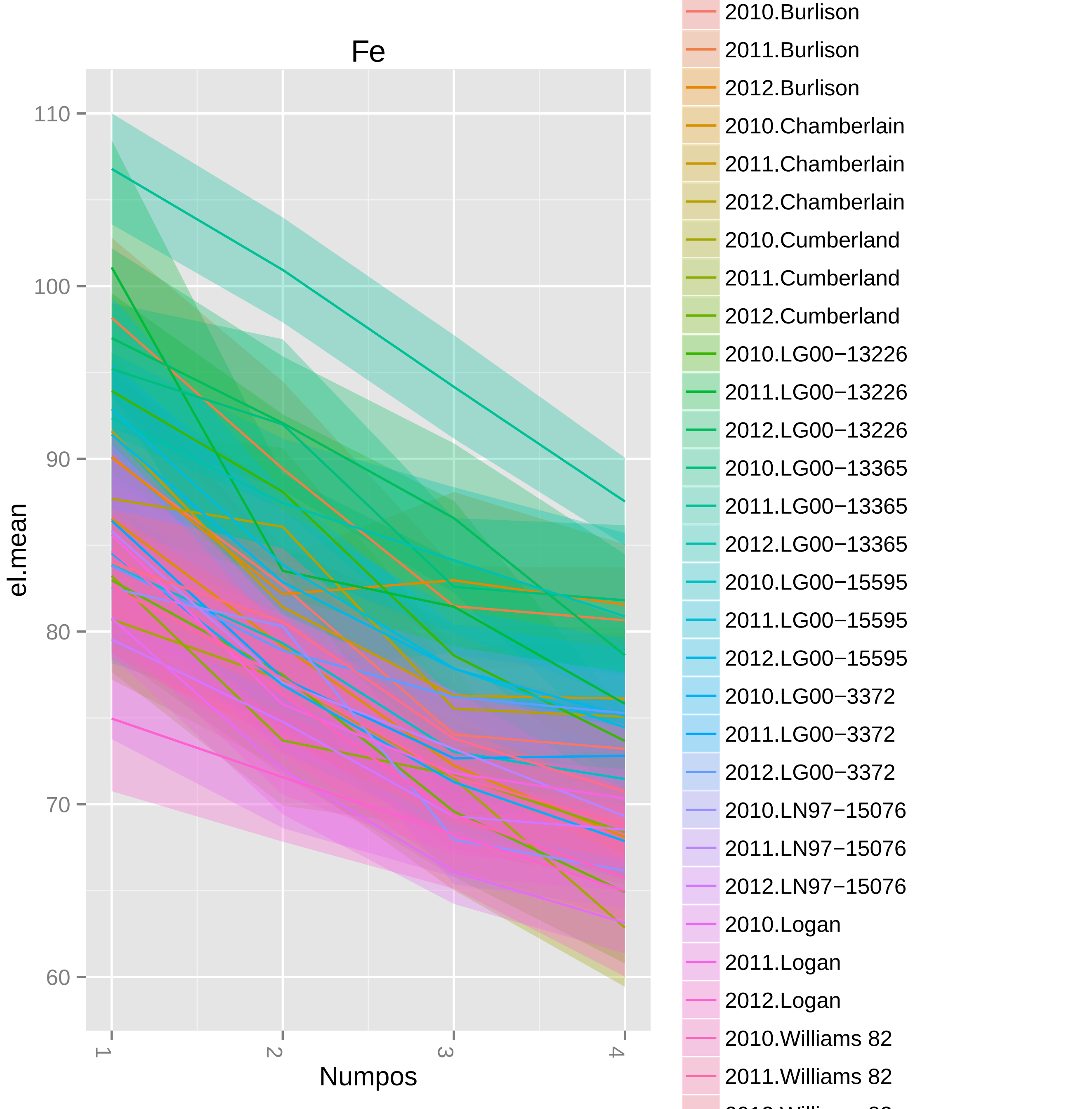
Fe
el.mean
110
100
90
80
70
60
Numpos
1
2
3
4
2010.Burlison
2011.Burlison
2012.Burlison
2010.Chamberlain
2011.Chamberlain
2012.Chamberlain
2010.Cumberland
2011.Cumberland
2012.Cumberland
2010.LG00−13226
2011.LG00−13226
2012.LG00−13226
2010.LG00−13365
2011.LG00−13365
2012.LG00−13365
2010.LG00−15595
2011.LG00−15595
2012.LG00−15595
2010.LG00−3372
2011.LG00−3372
2012.LG00−3372
2010.LN97−15076
2011.LN97−15076
2012.LN97−15076
2010.Logan
2011.Logan
2012.Logan
2010.Williams 82
2011.Williams 82

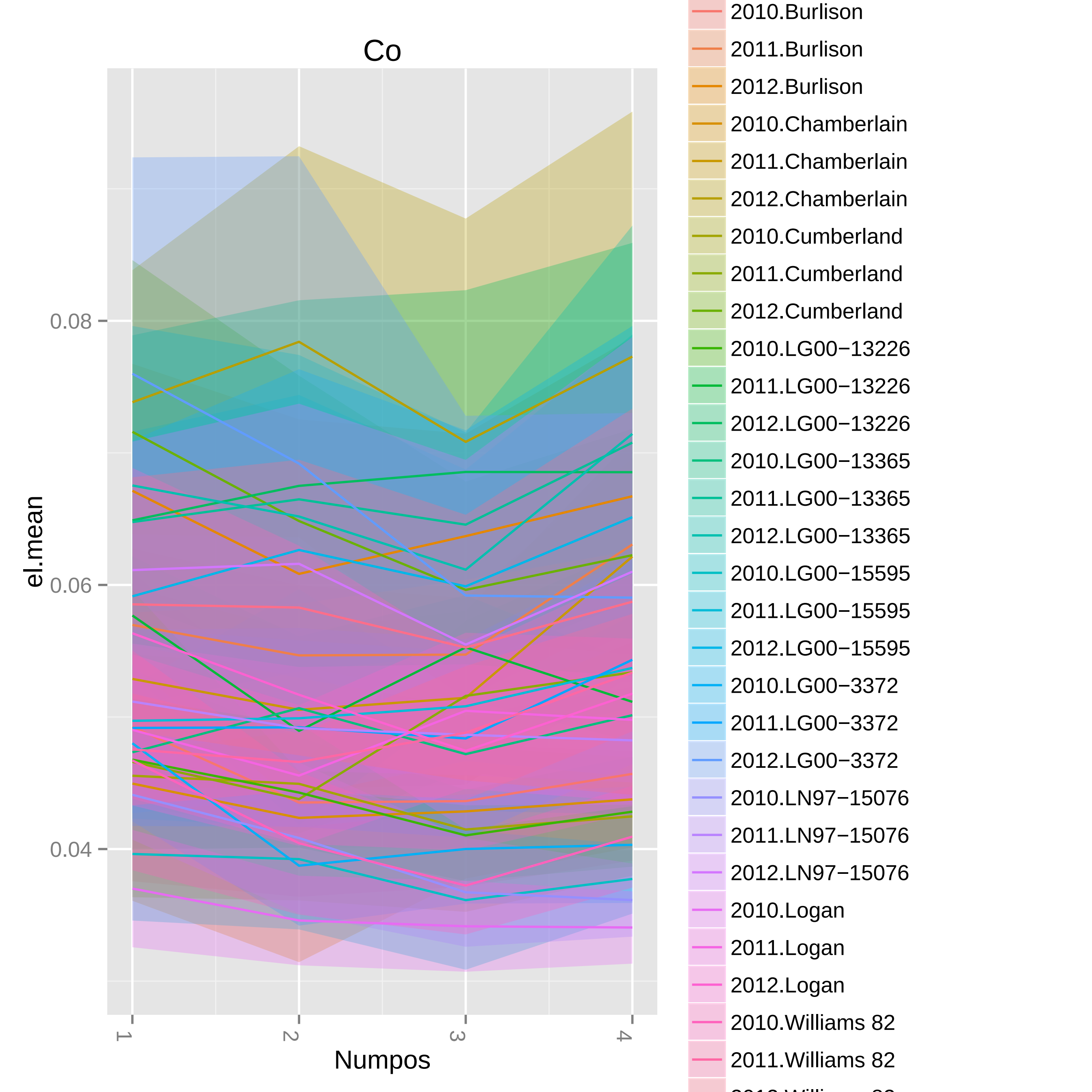
Co
el.mean
0.08
0.06
0.04
Numpos
1
2
3
4
2010.Burlison
2011.Burlison
2012.Burlison
2010.Chamberlain
2011.Chamberlain
2012.Chamberlain
2010.Cumberland
2011.Cumberland
2012.Cumberland
2010.LG00−13226
2011.LG00−13226
2012.LG00−13226
2010.LG00−13365
2011.LG00−13365
2012.LG00−13365
2010.LG00−15595
2011.LG00−15595
2012.LG00−15595
2010.LG00−3372
2011.LG00−3372
2012.LG00−3372
2010.LN97−15076
2011.LN97−15076
2012.LN97−15076
2010.Logan
2011.Logan
2012.Logan
2010.Williams 82
2011.Williams 82

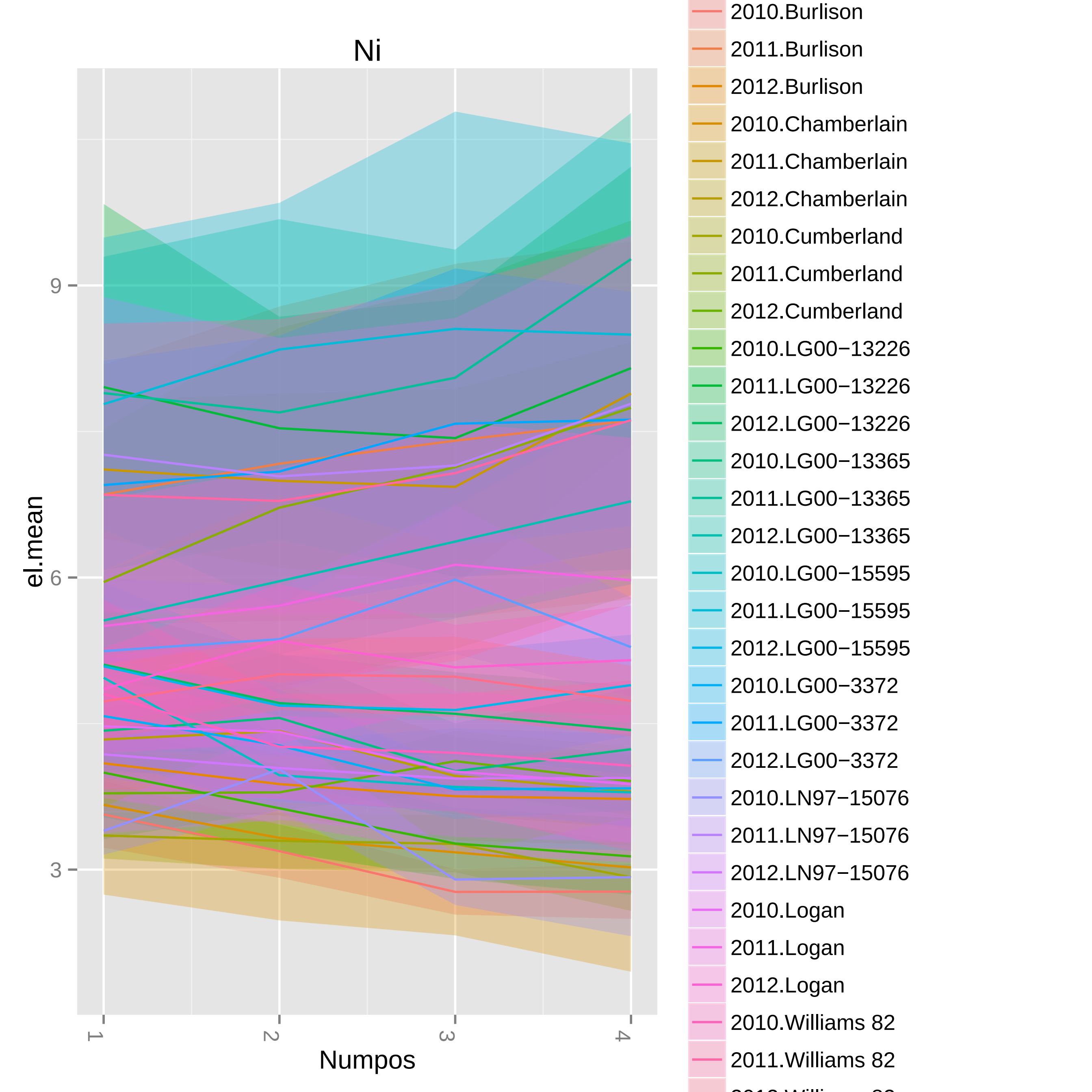
Ni
el.mean
9
6
3
Numpos
1
2
3
4
2010.Burlison
2011.Burlison
2012.Burlison
2010.Chamberlain
2011.Chamberlain
2012.Chamberlain
2010.Cumberland
2011.Cumberland
2012.Cumberland
2010.LG00−13226
2011.LG00−13226
2012.LG00−13226
2010.LG00−13365
2011.LG00−13365
2012.LG00−13365
2010.LG00−15595
2011.LG00−15595
2012.LG00−15595
2010.LG00−3372
2011.LG00−3372
2012.LG00−3372
2010.LN97−15076
2011.LN97−15076
2012.LN97−15076
2010.Logan
2011.Logan
2012.Logan
2010.Williams 82
2011.Williams 82

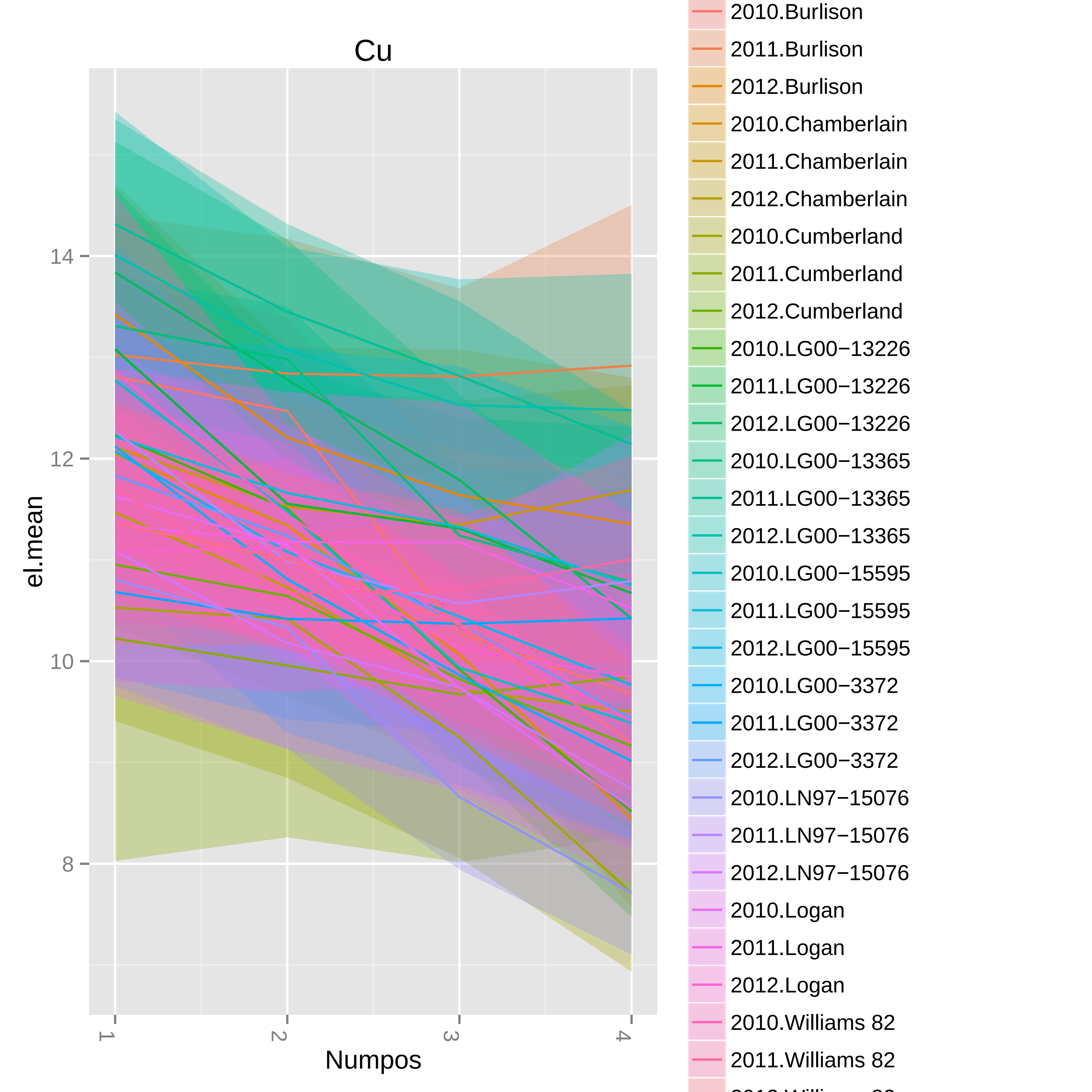
Cu
el.mean
14
12
10
8
1
2
3
4
Numpos
2010.Burlison
2011.Burlison
2012.Burlison
2010.Chamberlain
2011.Chamberlain
2012.Chamberlain
2010.Cumberland
2011.Cumberland
2012.Cumberland
2010.LG00−13226
2011.LG00−13226
2012.LG00−13226
2010.LG00−13365
2011.LG00−13365
2012.LG00−13365
2010.LG00−15595
2011.LG00−15595
2012.LG00−15595
2010.LG00−3372
2011.LG00−3372
2012.LG00−3372
2010.LN97−15076
2011.LN97−15076
2012.LN97−15076
2010.Logan
2011.Logan
2012.Logan
2010.Williams 82
2011.Williams 82

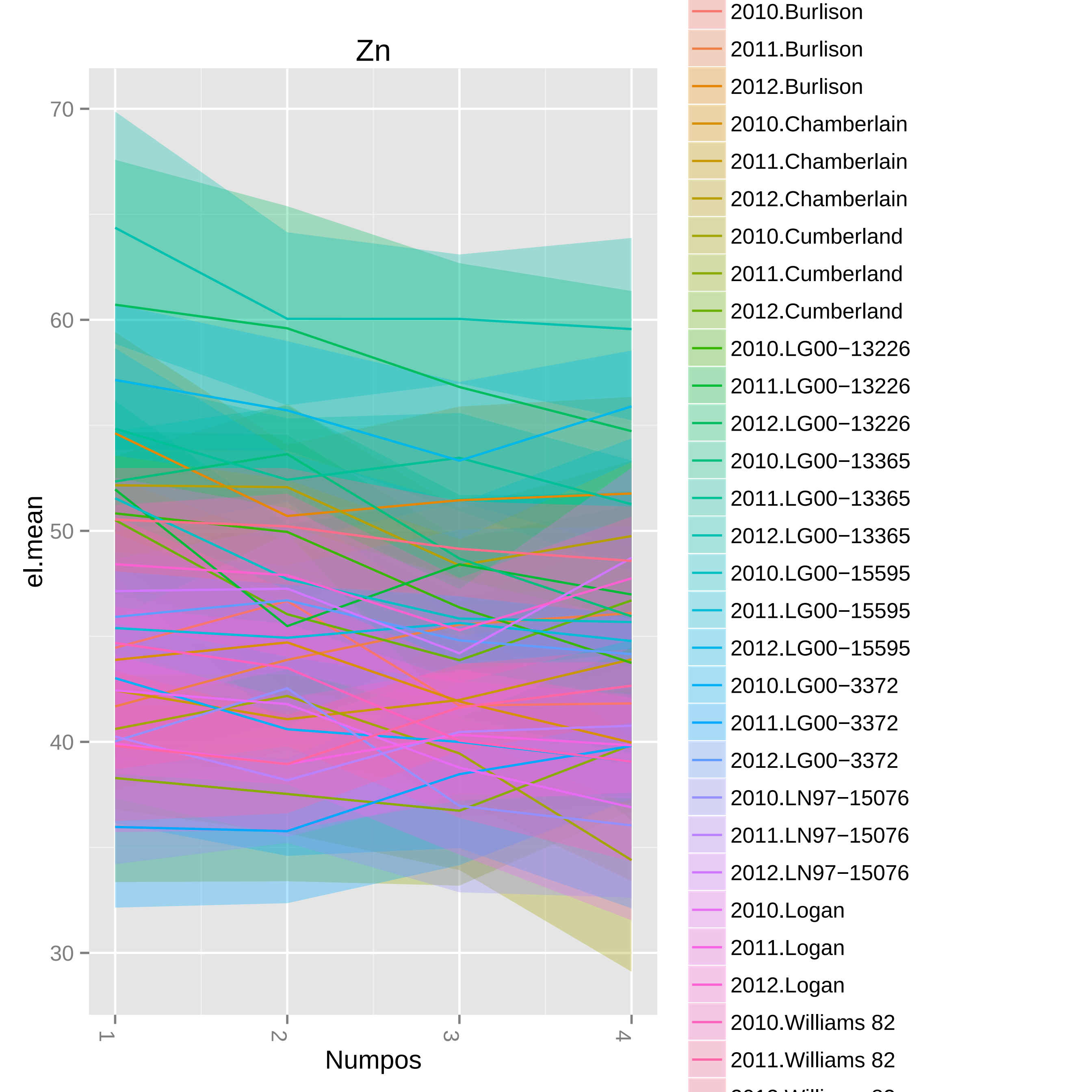
Zn
el.mean
70
60
50
40
30
1
2
3
4
Numpos
2010.Burlison
2011.Burlison
2012.Burlison
2010.Chamberlain
2011.Chamberlain
2012.Chamberlain
2010.Cumberland
2011.Cumberland
2012.Cumberland
2010.LG00−13226
2011.LG00−13226
2012.LG00−13226
2010.LG00−13365
2011.LG00−13365
2012.LG00−13365
2010.LG00−15595
2011.LG00−15595
2012.LG00−15595
2010.LG00−3372
2011.LG00−3372
2012.LG00−3372
2010.LN97−15076
2011.LN97−15076
2012.LN97−15076
2010.Logan
2011.Logan
2012.Logan
2010.Williams 82
2011.Williams 82
