# Supplementary figures and images for: Canopy position has a profound effect on soybean seed composition
Source: PeerJ. 2016 Sep 13;4:e2452. doi: 10.7717/peerj.2452 (PMC5028787; doi:10.7717/peerj.2452)

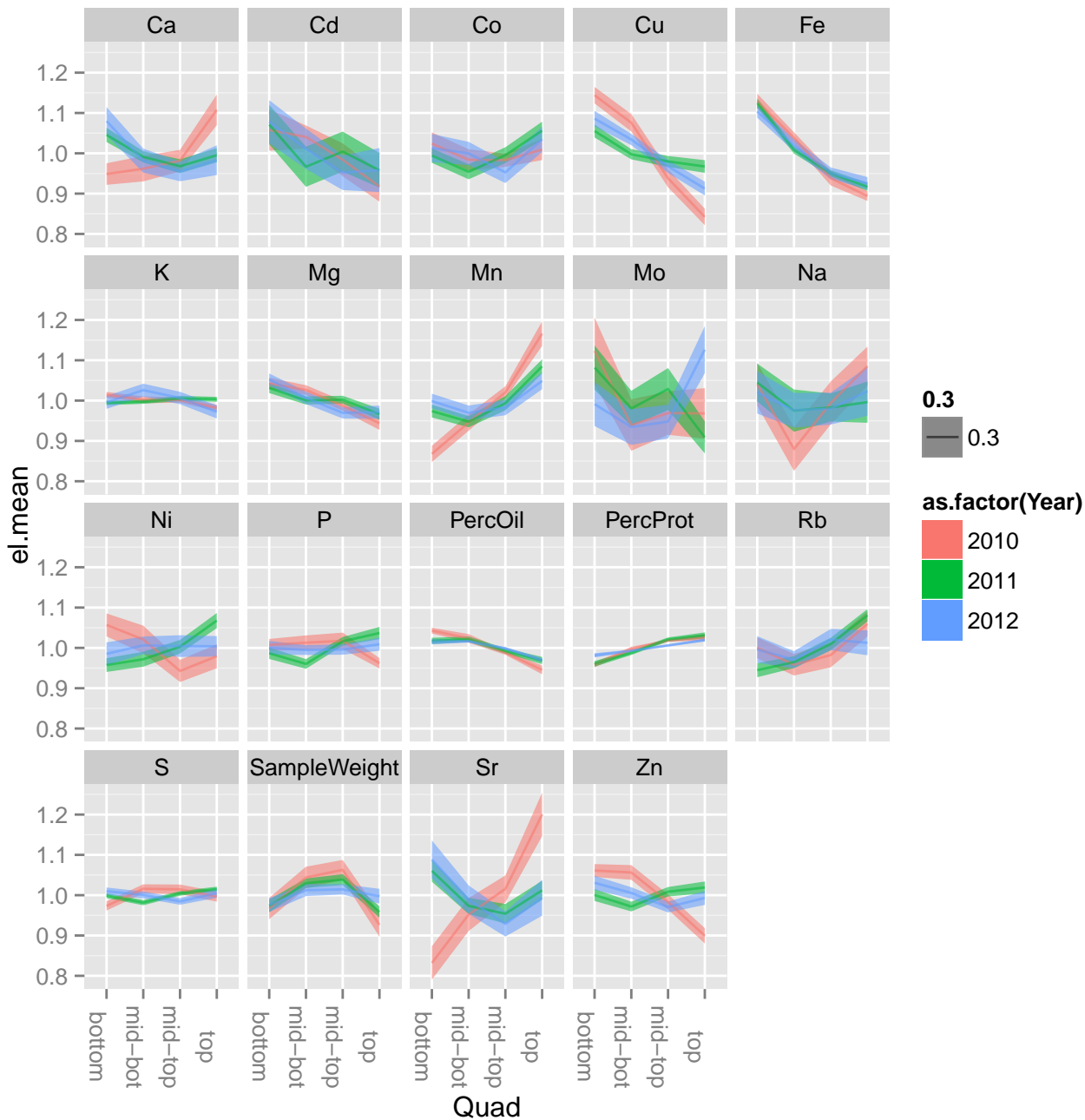

Supplement: File S2 [file peerj-04-2452-s002.pdf]

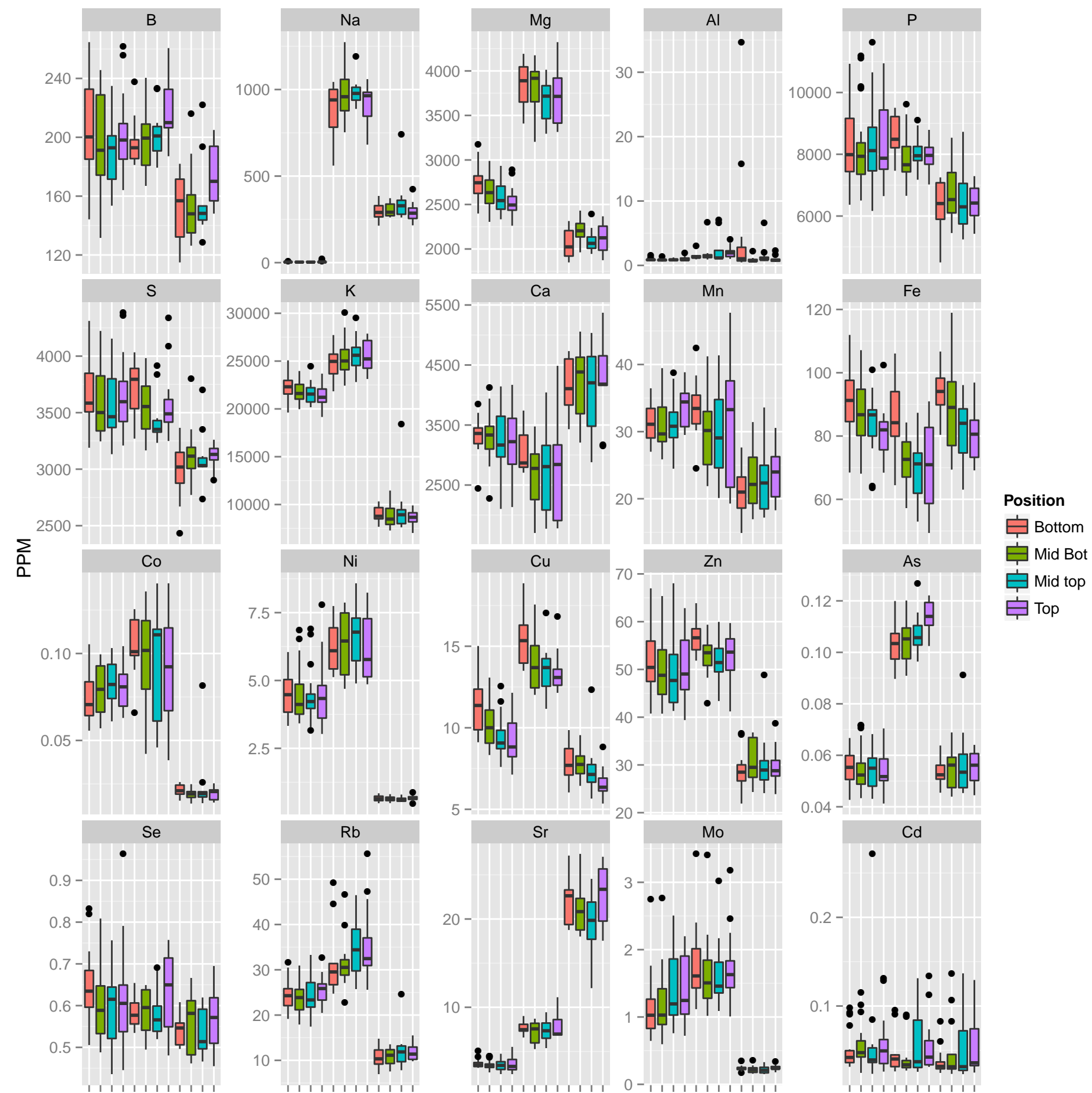

Supplement: File S6 [file peerj-04-2452-s006.pdf]

## Supplemental File 7

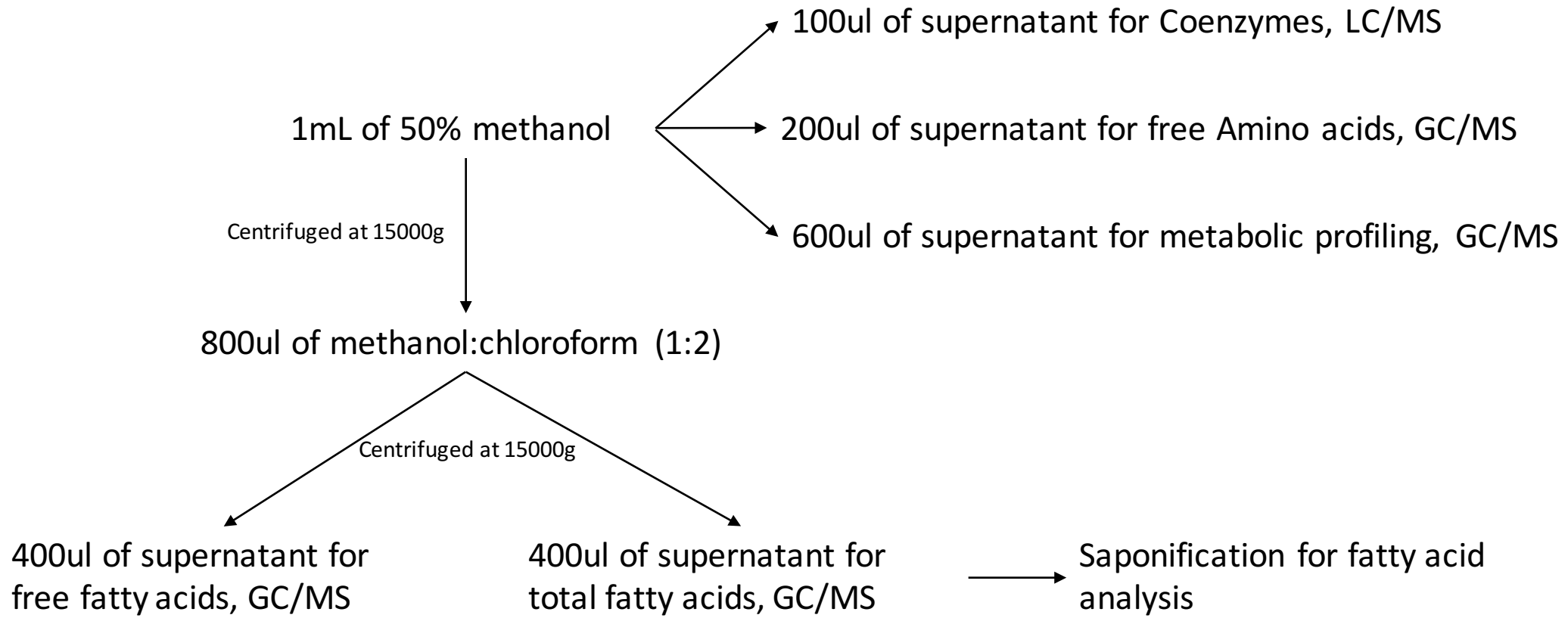

Supplement: File S7 [file peerj-04-2452-s007.pdf]

A

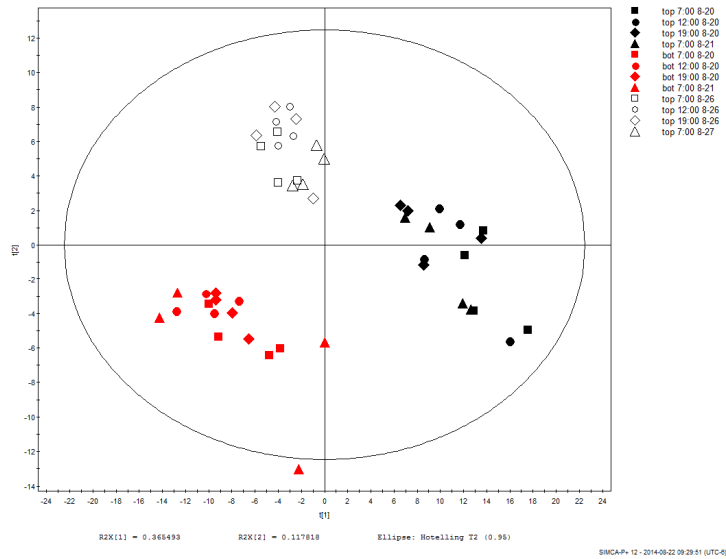

Autoscale and log transformation for A) and B) row normalization for C).

B

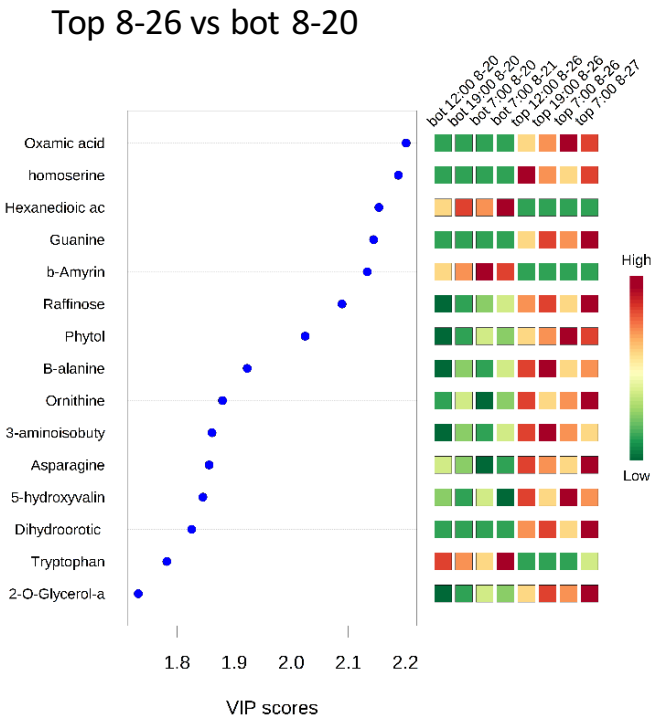

Supplement: File S8 — (A) PLS-DA scores plot (R2 = 98.7%, Q2 = 81.1%, P < 0.001 by permutation test) of soybean seeds at different canopy position and time of day. (B) Variable Importance in the Projection (VIP) for the first component showing the fifteen most important compounds. [file peerj-04-2452-s008.pdf]
