# Supplementary material for: Canopy position has a profound effect on soybean seed composition
Source: PeerJ. 2016 Sep 13;4:e2452. doi: 10.7717/peerj.2452 (PMC5028787; doi:10.7717/peerj.2452)
Supplement: Table S1 [file peerj-04-2452-s009.docx]

**Supplemental Table 1**

Indeterminate lines used in the present study

| **Genotype** | **MG** | **Pedigree** | **Protein** | **%PRO^a^** | **%OIL^a^** | **Comments** |
| --- | --- | --- | --- | --- | --- | --- |
| Burlison | II | K74-113-76-486 x Century (K74-113-76-486 is Tracy x Pomona) | medium | 36.4 | 17.8 |  |
| Chamberlain | III | A76-304020 x Land O Lakes Max (A76-304020 is from (Beeson x AP68-1016) x (L15 x Calland) AP68-1016 is from Clark(5) x PI 84946-2 L15 is from Wayne (6) x Clark 63), Max is from [Wayne x (Clark x Adams)] x Cutler | normal | 32.5 | 18.8 |  |
| Cumberland | III | Corsoy x Williams | normal | 32.8 | 19.8 |  |
| LG00-13226 | III | PI 437088A x Burlison | high | 41.5 | 15.2 | Derived from exotic germplasm |
| LG00-3372 | III | PI 561319A x PI 574477 | normal | 32.1 | 18.7 | Derived from exotic germplasm |
| LG00-13365 | II | Williams 82 x PI 437088A | high | 40.4 | 15.8 | Derived from exotic germplasm |
| LG00-15595 | III | Williams 82 (3) x Wisconsin Black | medium | 37.1 | 17.3 | Derived from exotic germplasm |
| LN97-15076 | IV | Macon x Stressland | normal | 32.4 | 18.4 |  |
| Logan | III | [Beeson x L15 (Wayne (6) x Clark 63)] x Amsoy | normal | 31.9 | 20.6 |  |
| Williams 82 | III | Williams (7) x Kingwa | normal | 33.6 | 18.7 |  |

^a^Average values for protein and oil from bottom, mid-bottom, mid-top, and top quadrants from 2012 based on 13% moisture
