# Supplementary material for: Canopy position has a profound effect on soybean seed composition
Source: PeerJ. 2016 Sep 13;4:e2452. doi: 10.7717/peerj.2452 (PMC5028787; doi:10.7717/peerj.2452)
Supplement: Table S2 [file peerj-04-2452-s010.docx]

**Figure 1—table supplement 2**

Weather summary (June 1– August 31) during the 2010 to 2012 growing seasons

| **Year** | **Temperature** | **Precipitation** | **Comments** |
| --- | --- | --- | --- |
| 2010^a^ | Above average | Above average | Generally warm spring and record low temperatures set during summer; some severe weather episodes. |
| 2011^b^ | Above average | Below average | Hot and humid during July |
| 2012^c^ | Above average | Below average | Drought conditions during late spring and summer, impacting crop growth. |

^a^http://www.crh.noaa.gov/ilx/?n=2010review

^b^http://www.crh.noaa.gov/ilx/?n=2011review

^c^http://www.crh.noaa.gov/ilx/?n=2012review
