# Supplementary material for: Canopy position has a profound effect on soybean seed composition
Source: PeerJ. 2016 Sep 13;4:e2452. doi: 10.7717/peerj.2452 (PMC5028787; doi:10.7717/peerj.2452)
Supplement: Table S3 [file peerj-04-2452-s011.docx]

**Supplemental Table 3**

Genotype differences in Seed fill period (SFP) and the difference in SFP at two node positions (bottom minus top; delSFP)

In 2012, each line was grown in rows with East-West or North-South orientation and values for each are shown and were used to calculate average values for each node position.
